# Clinical Outcomes of an Innovative Cefazolin Delivery Program for MSSA Infections in OPAT

**DOI:** 10.3390/jcm11061551

**Published:** 2022-03-11

**Authors:** Laura Herrera-Hidalgo, Rafael Luque-Márquez, Aristides de Alarcon, Ana Belén Guisado-Gil, Belen Gutierrez-Gutierrez, Maria Dolores Navarro-Amuedo, Julia Praena-Segovia, Juan Manuel Carmona-Caballero, Elena Fraile-Ramos, Alicia Gutierrez-Valencia, Luis Eduardo Lopez-Cortes, Maria Victoria Gil-Navarro

**Affiliations:** 1 Unidad de Farmacia, Hospital Universitario Virgen del Rocío, 41013 Seville, Spain; laura.herrera.sspa@juntadeandalucia.es (L.H.-H.); anab.guisado.sspa@juntadeandalucia.es (A.B.G.-G.); mariav.gil.sspa@juntadeandalucia.es (M.V.G.-N.); 2 Instituto de Biomedicina de Sevilla (IBiS), 41013 Seville, Spain; rafael.luque.sspa@juntadeandalucia.es (R.L.-M.); caristo.alarcon.sspa@juntadeanalucia.es (A.d.A.); belen.gutierrez.sspa@juntadeandalucia.es (B.G.-G.); mdolores.navarro.sspa@juntadeandalucia.es (M.D.N.-A.); julia.praena.sspa@juntadeandalucia.es (J.P.-S.); juanm.caballero.sspa@juntadeandalucia.es (J.M.C.-C.); elena.fraile.sspa@juntadeandalucia.es (E.F.-R.); agutierrez-ibis@us.es (A.G.-V.); 3 CSIC—Consejo Superior de Investigaciones Científicas, 28006 Madrid, Spain; 4 Unidad Clínica de Enfermedades Infecciosas, Microbiología y Medicina Preventiva, Hospital Universitario Virgen del Rocío, 41013 Seville, Spain; 5 Centro de Investigación Biomédica en Red de Enfermedades Infecciosas, 28029 Madrid, Spain; 6 Unidad Clínica de Enfermedades Infecciosas, Microbiología y Medicina Preventiva, Hospital Universitario Virgen Macarena, 41009 Seville, Spain

**Keywords:** methicillin-susceptible *Staphylococcus aureus*, MSSA, OPAT, cefazolin, home-delivery

## Abstract

Cefazolin is a recommended treatment for methicillin-susceptible *Staphylococcus aureus* (MSSA) infections that has been successfully used in outpatient parenteral antibiotic therapy (OPAT) programs. The aim of this study was to assess the clinical outcomes of cefazolin delivered each day (Group 24) vs. every two days (Group 48) for MSSA infections in OPAT programs. It was a prospective observational study with retrospective analysis of a cohort of MSSA infections attended in OPAT. The primary outcome was treatment success, defined as completing the antimicrobial regimen without death, treatment discontinuation, or readmission during treatment and follow-up. A univariate and multivariate logistic regression model was built. A two-sided *p* < 0.05 was considered statistically significant. Of the 149 MSSA infections treated with cefazolin 2 g/8 h in OPATs, 94 and 55 patients were included in the delivery Group 24 and Group 48, respectively. Treatment failure and unplanned readmission rates were similar in both groups (11.7% vs. 7.3% *p* = 0.752 and 8.5% vs. 5.5% *p* = 0.491). There was a significant increase in vascular access complications in Group 24 (33.0%) with respect to Group 48 (7.3%) (*p* < 0.001). Treating uncomplicated MSSA infection with cefazolin home-delivered every two days through an OPAT program is not associated with an increased risk of treatment failure and entails a significant reduction in resource consumption compared to daily delivery.

## 1. Introduction

Methicillin-susceptible *Staphylococcus aureus* (MSSA) is a pathogen that causes serious nosocomial and community-acquired infections [1], with high morbidity and mortality [2]. The standard therapy for MSSA infections is anti-staphylococcal penicillin (oxacillin, flucloxacillin, or nafcillin), although several studies have reported similar efficacy with cefazolin with fewer adverse events [3,4].

Outpatient parenteral antimicrobial therapy (OPAT) allows patients to complete the antimicrobial treatment at home. The safety and effectiveness of OPAT have been extensively demonstrated [5]. Most of the studies have also shown that these programs are cost-effective [6] and associated with greater comfort for the patient and a lower risk of nosocomial complications [7]. Patients with MSSA infections often involve long-term inpatient antimicrobial therapy; therefore, OPAT is a highly attractive alternative for these patients.

A variety of OPAT models have been described, including home-based and clinic-based programs and a self-administration model [8,9]. The resources required in each model are different and determine the number and the type of patients included. In Europe, a frequent OPAT model is the one based on daily nurse visitation at the patient’s home together with an infectious disease physician overview and follow-up. Usually, nurse visitations are scheduled daily and include drug administration and patient evaluation [7,10]. The main advantages of this model are the continuous supervision of the patient by a health care professional, the early detection of adverse drug effects or infection progression, and the guaranteed continuity of care by the same health care team. In addition, this model prevents the overcharge caused by the hiring of external companies typical of the other OPAT models. This OPAT program requires the daily involvement of the hospital pharmacy for drug preparation, and the antibiotics delivered daily are already diluted. Drug administration as a bolus, intermittent infusion, or continuous infusion depends on their pharmacodynamic and pharmacokinetic profile, solution stability, and the administration resources available in each OPAT program, such as elastomeric pumps or electronic infusion pumps [8,9,10,11].

Cefazolin and anti-staphylococcal penicillins have been successfully used in OPAT [12,13,14,15,16]. Some of the major benefits of cefazolin are its association with significant reductions in nephrotoxicity and hepatotoxicity, lower likelihoods of phlebitis and hypersensitivity reactions, and discontinuation due to side effects [17,18]. One study has demonstrated that twice-daily cefazolin for serious MSSA infection in OPAT was safe and effective. However, twice-daily nurse visitation entails more human resources and limits its utility [13]. Other programs deliver cefazolin via an electronic pump, administering 2 g every 8 h, reducing nurse home visitation to once daily [16]. Cefazolin chemical stability at room temperature for at least 5 days was previously proved [19,20], whereas it wasn’t until 2019 that our OPAT program policy considered cefazolin delivery every two days. The change in policy was motivated by the need for the optimization of OPAT resources. Therefore, leveraging its stability, cefazolin was prepared and delivered every two days and administered for 48 h through an electronic pump, which was programmed to release 2 g every 8 h. Thus, it allowed for reducing nurse visitation and pharmacy preparation to every two days. This new model of delivery maintains the continuous supervision of the patient by a health care team of our traditional OPAT model, whereas it allows twice as many patients to be treated without increasing the resources needed, especially pharmacy and nurse workload. The spread of this practice would entail significant savings for health care systems and enable more patients to benefit from the advantages of an OPAT program.

The aim of this study was to assess the clinical outcomes of cefazolin delivered daily vs. every two days for MSSA infections in OPAT programs.

## 2. Materials and Methods

### 2.1. Study Design and Setting

We conducted a prospective observational study with retrospective analysis of a cohort of patients attended in an OPAT program shared by two tertiary teaching hospitals. The main characteristics of our OPAT program have been described elsewhere [21]. Briefly, patients without oral or intramuscular antibiotic alternatives were included in the OPAT program. The antimicrobial treatment and patient inclusion criteria in the OPAT program were settled on by a multidisciplinary team. An infectious disease physician oversaw the patient selection. Patients included in hemodialysis programs were not included in our program. The OPAT program included home visitation by the nurse team for drug administration and clinical care and weekly reviews by an infectious diseases physician. After completion of the antibiotic treatment, a one-month follow-up was established for uncomplicated infections (i.e., uncomplicated bacteremia) and a 6-month to a 1-year follow-up was established for complicated or deep-seated infections (i.e., infective endocarditis). All antibiotic treatments were prepared by the pharmacy service under sterile conditions. The STROBE guidelines for reporting observational studies were followed [22]. This program has the approval of the Ethics Committee for Clinical Research of the two hospitals involved.

### 2.2. Patient Population

Eligible patients for the current study included patients with culture-confirmed MSSA infections treated with 2 g of cefazolin every 8 h (except for renal impairment adjustments) as a continuation regimen between July 2012 and August 2021. Treatments were delivered daily (6 g) or every two days (12 g), in both cases, the cefazolin concentration was 24 mg/mL, and it was administered in a bolus (2 g/8 h). Assignation of the groups of treatment (daily delivery or delivery every 48 h) was at the discretion of the clinician and the resources available.

### 2.3. Data Collection and Definitions

The following data were recorded in a preexisting OPAT database: sex, age, Charlson comorbidity index, comorbidities, diagnosis infection, type of vascular access, infection acquisition, duration of treatment (inpatient treatment and by OPAT), clinical outcomes, and adverse events. The primary sources of infection were defined according to the Centers for Disease Control and Prevention [23]. Uncomplicated MSSA bacteriemia [24,25] and the type of acquisition [26,27] were considered according to previously defined criteria The primary outcome was treatment success, defined as completing the antimicrobial regimen without death, treatment discontinuation, or all-cause readmission during treatment or follow-up. Treatment failure was defined as unfavorable clinical course, infection relapse or progression, death, unplanned readmission, or premature treatment interruption. The secondary outcomes were unplanned readmission rates during follow-up, adverse events, and vascular access complications.

### 2.4. Statistical Analysis

Firstly, a descriptive analysis was performed considering the baseline characteristics as well as the primary and secondary outcomes. Categorical variables were summarized as percentages. Continuous variables were summarized as the median and interquartile range (IQR). Quantitative variables were compared using the Mann–Whitney test, and categorical variables were compared using the chi-square test or Fisher exact *t* test.

Secondly, a univariate and multivariate logistic regression model was built to determine the factors associated with treatment failure, estimating odds ratios (ORs) and 95% confidence intervals (CIs). We included all the explicative variables that are clinically relevant or that have already been identified as being associated with poor outcomes in the univariate analysis—age, Charlson score, cancer, chronic renal failure, acquisition of the infection, type of infection, inpatient treatment, vascular access, vascular access complication, and treatment group. No data were missing in the multivariate model.

A two-sided *p* < 0.05 was considered statistically significant. The statistical analysis was performed using SPSS version 28.0 (SPSS Inc., Chicago, IL, USA) and R 3.6.1 software (https://www.r-project.org/, accessed on 22 December 2021).

## 3. Results

### 3.1. Study Population Characteristics

**Figure 1:**
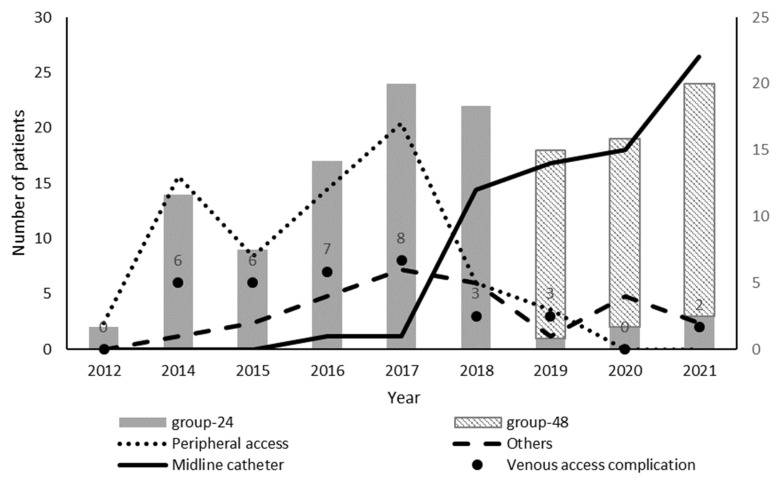
Evolution of patient inclusion in each delivery group and vascular access type and complications over the years.

Of the 1595 patients included in the OPAT program, 149 had MSSA infections treated with cefazolin as a continuation regimen. Among them, daily delivery (Group 24) was arranged for 94 patients and delivery every 48 h (Group 48) for 55 patients. Until 2019, all treatments were delivered daily (Figure 1). The patients’ baseline characteristics are shown in Table 1. In this cohort, the most frequent comorbidities were cancer (37.6%), cardiac insufficiency (33.6%), and diabetes mellitus (29.5%). Cancer and renal chronic renal failure prevalence were significantly different among the treatment groups. Totals of 29 (30.9%) and 27 (49.1%) patients were diagnosed with cancer at the time of their admission into OPAT in Group 24 and Group 48, respectively (*p* = 0.027). Chronic renal failure was detected in 20 (21.3%) and 4 (7.3%) patients in Group 24 and Group 48, respectively (*p* = 0.025).

Several MSSA infections were included in this cohort (Table 1). The most common was catheter-related bloodstream infection (44.4%), which accounted for 41.5% and 49.1% of patients in the OPAT courses in Group 24 and Group 48, respectively; followed by primary bacteriemia (12.1%). Other infections, such as prosthetic articular joint infection, septic arthritis, and infective endocarditis, were mainly treated in Group 24 (9 vs. 2, 6 vs. 1, and 5 vs. 0, respectively). Nevertheless, globally, no statistically significant differences were found in the diagnosis distribution among the treatment groups (*p* = 0.078). No differences were found between the groups in the proportions of non-bacteremic infections, uncomplicated bacteremia, and complicated bacteremia (*p* = 0.412).

### 3.2. MSSA Treatment

The median duration of the inpatient treatment was similar in both groups, with 7 (4–10) days in Group 24 and 7 (6–13) days in Group 48 (*p* = 0.208) (Table 2). Cefazolin was the antimicrobial most commonly prescribed as inpatient treatment (77.9%), followed by cloxacillin (12.1%). The cefazolin inpatient treatment was less frequent in Group 24 compared to Group 48 (71.3% vs. 89.1%) (*p* = 0.036). The median duration of the OPAT course was 9 (5–13) and 10 (7–16) days in Group 24 and Group 48 (*p* = 0.163), respectively. The most common type of venous access was peripheral access (59.6%) in Group 24 and midline catheter (81.8%) in Group 48 (*p* < 0.000). The timeline of vascular access utilization is shown in Figure 1.

### 3.3. Clinical Outcomes

The main clinical outcomes are summarized in Table 2. The rates of unplanned readmission and treatment failure in Group 24 and Group 48 were 7.4% (*n* = 11) and 10.1% (*n* = 15), respectively. Among the patients treated in Group 24, the causes of treatment failure (11.7%) included infection relapse or progression (*n* = 6), death (*n* = 3), antibiotic side effects (*n* = 1), and lack of familial support (*n* = 1). Eight patients (8.5%) required unplanned hospitalization; seven were related to infection relapse or progression (one of them died) and one due to a lack of familial support. Two deaths took place at the patients’ homes, one as a result of infection progression with palliative support and one presumably because of a malignant arrhythmia. The third patient died at the hospital, after readmission resulting from infection progression. The diagnoses of the three patients who died in this group were primary bacteremia in two cases and infective endocarditis in one case. Regarding Group 48, the unplanned readmission rate was 5.5% (*n* = 3), in all cases because of infection progression. In this group, treatment failure was detected in four (7.3%) patients, three of them due to an unfavorable clinical course and one because of the death of the patient, diagnosed with cholangitis, after palliative care at home. No differences were found regarding treatment failure, unplanned readmission, or death between the groups.

### 3.4. Univariate and Multivariable Analysis Model

**Figure 2:**
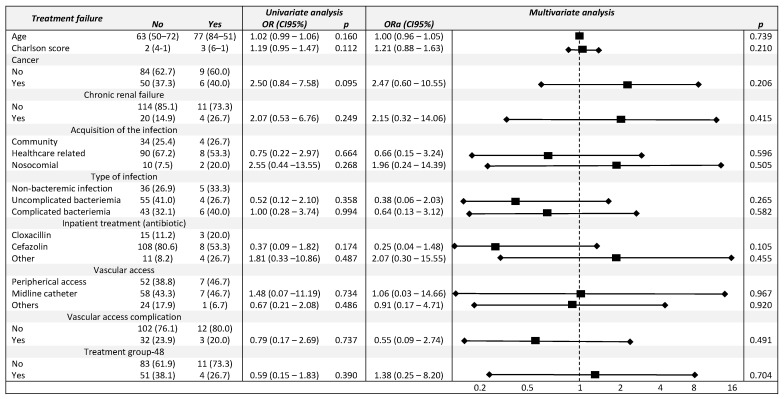
Factors related to treatment failure. Univariate and multivariate analysis. OR = odds ratios; Ora = adjusted OR (represented by squares), CI 95% = 95% confidence intervals (represented by lines ended with rhombus).

Treatment in Group 48 was not associated with higher treatment failure than Group 24 in univariate analysis (unadjusted OR, 0.59; 95% CI, 0.15–1.83; *p* = 0.390). In addition, after adjusting for potential confounding factors, treatment failure was not found to be different between Group 24 and Group 48 (adjusted OR (ORa), 1.38; 95% CI, 0.25–8.20; *p* = 0.704). None of the variables included in the multivariate analysis were significantly associated with treatment failure (Figure 2). The rate of vascular access complication was 23.3% in the groups. There was a significant increase in these complications in Group 24 (33.0%) with respect to Group 48 (7.3%) (*p* < 0.001). The evolution of vascular access complication over the years is shown in Figure 1. The main cause was extravasation, malfunction, or catheter loss in both groups (74.2% vs. 100%). Phlebitis occurred in 8 patients, all in Group 24.

## 4. Discussion

Cefazolin is a widely recommended treatment for MSSA infections, endorsed by good clinical outcomes, lower rates of adverse events, and a reduced number of administrations compared to anti-staphylococcal penicillins [4,12,16]. To the best of our knowledge, this is the first study describing and analyzing the implementation of an innovative cefazolin delivery OPAT program. In our experience, similar treatment success rates have been observed with 24 h and 48 h delivery programs. This delivery option allows twice as many patients to be treated.

Cefazolin delivery through OPAT has been previously described using a wide variety of OPAT models [12,13,14,15,16]. Nurse interventions ranged between pre-discharge training in self-administration modality and twice-daily home visitations. Compared to the home- or clinical-based experiences previously reported, our 48 h delivery program reduced the need for nurse intervention for drug administration and catheter manipulations by 50–75%. In these studies, cefazolin treatment failure and unplanned readmission for MSSA infections oscillated between 6.7 and 32.1%, and 9 and 21%, respectively [12,13,14,15,16]. In our cohort, both treatment failure and unplanned readmission rates were low (10.1% and 7.4%) and similar in both groups. In addition, our antimicrobial adverse events rate was reduced (0.67%) compared to those previously reported (11.7–4.0%) [12,13,14,15,16]. The low incidence of vascular access complication (7.3%) in the 48 h delivery cohort should be analyzed. It was remarkably lower than the rate found in the 24 h delivery group (33.0%) and others previously reported in OPAT (19.1%) [28]. However, it might be explained by the predominant use of midline catheters, rather than by the reduction in catheter manipulations.

During the last decade, ceftriaxone, a drug with a pharmacokinetic profile that allows for a single daily administration, has been proposed as an alternative continuation treatment in OPAT for MSSA infections [14,15,29,30,31]. These observational studies, compared to the efficacy and safety of standard care treatment (cefazolin or anti-staphylococcal penicillins), with ceftriaxone treatment for MSSA infections and reporting heterogeneous results, must be considered with caution. Furthermore, regarding antimicrobial stewardship, OPAT programs are a powerful tool to promote the appropriate use of antimicrobials [32]. Ceftriaxone spectrum is broader than the standard care treatment for MSSA infections and its use should be avoided whenever possible due to their ability to select extended-spectrum beta-lactamase-producing *Enterobacterales* and the high risk of *Clostridioides difficile* infection [32,33].

New trends in OPAT are pointing towards reducing the consumption of health care resources [32,34]. Several strategies have been proposed in this scenario, such as the OPAT self-administration modality (S-OPAT) or treatment with long-acting agents. In S-OPAT, antibiotics are administered at home by the patient or caregiver, and it involves an initial training and periodic visitations. Patient adherence and minimal features required for safe home sterile infusion are critical issues of this model that reduce its applicability [8]. Long-acting antimicrobial agents, such as dalbavancin or oritavancin, have been occasionally used for MSSA infections [35]. Administration once weekly or every two weeks lessens the number of visitations needed and avoids a permanent venous access device. Nevertheless, the economic impact of these new antibiotics, their broader spectrum—generally saved for penicillin-resistant gram-positive microorganisms—and the scarcity of clinical data prevent them from being generally recommended for MSSA infections.

Lastly, the interest in oral stepdown therapy for *Staphylococcus aureus* bacteremia is increasing and several experiences have been reported in the last several years [36,37,38,39,40]. Potential benefits have been described for the oral treatment of uncomplicated *S. aureus* bacteremia, whereas the definition of uncomplicated bacteremia remains controversial. In addition, only low-grade evidence supported the effectiveness and safety of oral switch in this syndrome. The utility of oral therapy is being assessed in an unpublished clinical trial [41]. In addition, antibiotic selection and therapy duration warrant further investigation, depending on the susceptibility of the isolates and features of the patients [39,42]. Meanwhile, oral therapy for MSSA infection should be considered on a case-by-case basis, weighing the potential benefits and risks.

Our results may have been influenced by the low proportion of deep-seated MSSA infection among the patients included in Group 48. In addition, nearly half of the patients treated in this group were diagnosed with uncomplicated bacteremia (45.5%). Despite no differences were found regarding the severity of the infection among the groups, cefazolin delivery every 48 h might be considered an efficient and safe OPAT modality for MSSA uncomplicated infections, whereas the inclusion of more severe MSSA infections warrants further investigations.

The present study has several limitations. First, its observational and retrospective nature implies that the treatment selection was done at the discretion of the attending physician, and the patients were not treated according to a defined protocol. Inherent to this design is the risk of bias due to confounding by indication. Second, a relatively small number of patients were enrolled in the study. Third, the inoculum effect was not routinely studied despite its possible influence on clinical outcomes [43]. The main strength of this study is that patients were included in a real-life scenario by receiving two cefazolin delivery programs. All patients included in our OPAT program had close follow-up evaluations and nurse visitation every day or every two days to promote the early detection of any complication.

## 5. Conclusions

In summary, the results of this study suggest that treating uncomplicated MSSA infection with cefazolin home-delivered every two days through an OPAT program is not associated with an increased risk of treatment failure, relapse, or mortality compared to daily delivery. Furthermore, cefazolin delivered every two days using a midline catheter may reduce vascular access complications and entails a significant reduction in resource consumption compared to daily delivery. These results should encourage well-designed research to strengthen the evidence and enable this novel delivery strategy with cefazolin in OPAT.

## Figures and Tables

**Table 1:** Baseline and MSSA infection.

Baseline Characteristics	Overall (n = 149)	Treatment		*p*
		Group 24 (n = 94)	Group 48 (n = 55)	
Age (Median—IQR)	63 (74–50)	65 (77–52)	57 (44–70)	0.023
Male gender	104 (69.8)	66 (70.2)	38 (69.1)	0.886
Charlson score (Median—IQR)	2 (1–4)	2 (1–4)	2 (1–4)	0.570
Comorbidities				
Cancer	56 (37.6)	29 (30.9)	27 (49.1)	0.027
Cardiac insufficiency	50 (33.6)	35 (37.2)	15 (27.3)	0.214
Diabetes mellitus	44 (29.5)	32 (34.0)	12 (21.8)	0.114
Chronic obstructive pulmonary disease	27 (18.1)	19 (20.2)	8 (14.5)	0.386
Chronic renal failure	24 (16.1)	20 (21.3)	4 (7.3)	0.025
Liver disease	10 (6.7)	5 (5.3)	5 (9.1)	0.375
**MSSA infection**				
Acquisition of the infection				
Community acquired	38 (25.5)	27 (28.7)	11 (20.0)	0.087
Nosocomial acquisition	13 (8.7)	11 (11.7)	2 (3.6)	
Health care-associated infection	98 (65.8)	56 (59.6)	42 (76.4)	
Type of infection				
Non-bacteremic infection	41 (27.1)	29 (30.9)	12 (21.8)	0.412
Uncomplicated bacteriemia	59 (39.6)	34 (36.2)	25 (45.5)	
Complicated bacteriemia	49 (32.9)	31 (33.0)	18 (32.7)	
Diagnosis				
Catheter-related bloodstream infection	66 (44.4)	39 (41.5)	27 (49.1)	0.078
Primary bacteriemia	18 (12.1)	6 (6.4)	12 (21.8)	
Prosthetic articular joint infection	11 (7.4)	9 (9.6)	2 (3.6)	
Septic arthritis	7 (4.7)	6 (6.4)	1 (1.8)	
Endocarditis	5 (3.4)	5 (5.3)	0 (0.0)	
Osteomyelitis	5 (3.4)	2 (2.1)	3 (5.5)	
Pyelonephritis	5 (3.4)	3 (3.2)	2 (3.6)	
Skin and soft tissue infection	4 (2.7)	3 (3.2)	1 (1.8)	
Pneumonia	3 (2.0)	2 (2.1)	1 (1.8)	
Intraabdominal infection	3 (2.0)	3 (3.2)	0 (0.0)	
Diabetic foot infection	2 (1.3)	2 (2.1)	0 (0.0)	
Others	20 (13.4)	14 (14.9)	6 (10.9)	

OPAT = Outpatient parenteral antibiotic therapy; MSSA = Methicillin-susceptible *Staphylococcus aureus*.

**Table 2:** Treatment characteristics and clinical outcomes.

Treatment Characteristics	Overall (n = 149)	Treatment		*p*
		Group-24 (n = 94)	Group-48 (n = 55)	
Inpatient Treatment (Days)	7 (5–11)	7 (4–10)	7 (6–13)	0.208
Inpatient treatment (antibiotic)				
Cefazolin	116 (77.9)	67 (71.3)	49 (89.1)	0.036
Cloxacillin	18 (12.1)	14 (14.9)	4 (7.3)	
Other or unknown	15 (10.1)	13 (13.8)	2 (3.6)	
OPAT treatment (days)	9 (6–14)	9 (5–13)	10 (7–16)	0.163
Vascular access				
Peripheral access	59 (39.6)	56 (59.6)	3 (5.5)	<0.000
Midline catheter	65 (43.6)	20 (21.3)	45 (81.8)	
Central access with peripheral insertion	15 (10.1)	9 (9.6)	6 (10.9)	
Central access	6 (4.0)	6 (6.4)	0 (0.0)	
Reservoir	4 (2.7)	3 (3.2)	1 (1.8)	
**Clinical outcomes**				
Treatment success (composite endpoint)	134 (89.9)	83 (88.3)	51 (92.7)	0.386
Treatment failure (composite endpoint)	15 (10.1)	11 (11.7)	4 (7.3)	0.386
Cause of treatment failure (% of treatment failure)				
Infection relapse or progression	9 (60.0)	6 (54.5)	3 (75.0)	0.816
Death during OPAT or readmission	4 (26.7)	3 (27.3)	1 (25.0)	
Antibiotic side effects	1 (6.7)	1 (9.1)	0 (0.0)	
Lack of familiar support	1 (6.7)	1 (9.1)	0 (0.0)	
Unplanned readmission	11 (7.4)	8 (8.5)	3 (5.5)	0.491
Vascular access complication	35 (23.3)	31 (33.0)	4 (7.3)	<0.001
Type of complication (% of vascular access complication)				
Phlebitis	8 (23.5)	8 (26.7)	0 (0.0)	0.247
Extravasation, malfunction, or catheter loss	26 (76.5)	23 (74.2)	4 (100.0)	

OPAT = Outpatient parenteral antibiotic therapy.
